# Complete genome of *Vibrio harveyi* isolate K2014767 from the hepatopancreas of captive Caribbean spiny lobster (*Panulirus argus*)

**DOI:** 10.1128/mra.01156-23

**Published:** 2024-04-22

**Authors:** Divya Rose, Hannah Bennett, Jennifer Dill-Okubo, Ruth Francis-Floyd, Alvin C. Camus, Geoffrey C. Waldbieser, Matt J. Griffin

**Affiliations:** 1 Department of Pathobiology and Population Medicine, College of Veterinary Medicine, Mississippi State University, Stoneville, Mississippi, USA; 2 School of Forest, Fisheries, and Geomatics Sciences, University of Florida, Gainesville, Florida, USA; 3 College of Veterinary Medicine, University of Florida, Gainesville, Florida, USA; 4 Department of Large Animal Clinical Sciences, College of Veterinary Medicine, University of Florida, Gainesville, Florida, USA; 5 Department of Pathology, College of Veterinary Medicine, University of Georgia, Athens, Georgia, USA; 6 Warmwater Aquaculture Research Unit, Agricultural Research Service, United States Department of Agriculture, Stoneville, Mississippi, USA; University of Southern California, Los Angeles, California, USA

**Keywords:** *Vibrio harveyi*, Carribean spiny lobster, *Panulirus argus*, hepatopancreas necrosis

## Abstract

The complete genome sequence is reported for *Vibrio harveyi* isolate K2014767, isolated from a captive Caribbean spiny lobster (*Panulirus argus*) during a species-specific mortality event in a public display aquarium in the United States.

## ANNOUNCEMENT

The Caribbean spiny lobster (*Panulirus argus*) inhabits the Atlantic Ocean from North Carolina, USA to Brazil. The largest of the spiny lobsters, *P. argus* comprises the second-largest lobster fishery in the United States (1–5). *Vibrio harveyi* is autochthonous to marine habitats and a pathogen of wild and captive marine fish and invertebrates (6–8), with reports of *V. harveyi*-associated disease in shrimp, crabs, and lobster (9, 10).

Bennett et al. (11) reported repeated species-specific mortality events in captive *P. argus* inhabiting a multispecies tank in a public aquarium. Moribund and dead lobsters showed no outward signs of disease. One fresh dead individual was sampled for necropsy, revealing Gram-negative bacilli in the hepatopancreas identified as *V. harveyi*.

*V. harveyi* K2014767 obtained from the lobster necropsy was revived from cryogenic storage on tryptic soy agar plates supplemented with 5% sheep blood. An individual colony was expanded in porcine brain heart infusion broth and expanded overnight with shaking (200 rpm) at 28°C. An aliquot (3 mL) of expanded culture was concentrated (20,000 × *g*) and genomic DNA was isolated from the pellet (DNEasy PowerSoil Pro kit; Qiagen, Hilden, Germany). Libraries were prepared using the RAD004 kit (Oxford Nanopore Technologies, Oxford, UK) for sequencing on Flongle flow cells (FLO-FLG001; R9.4.1). No size selection was performed prior to library preparation. Base calling was performed with Guppy v4.4.0. Sequencing reads were trimmed (minimum quality: Q10; minimum length: 2 kb; N50: 18,253 bp) using Nanofilt (12) to produce 281 Mb in 24,696 reads. Reads were corrected, trimmed to 39.8× genome coverage and assembled using Canu v2.1.1 (13), yielding five contigs. Contig sequence quality was improved with Nanofilt-trimmed reads and one iteration of Medaka v1.2.3 (https://github.com/nanoporetech/medaka). Paired-end Illumina reads were generated from the same DNA sample at the Georgia Genomics and Bioinformatics Core Facility on a NextSeq platform. Raw Illumina paired reads were trimmed using Trimmomatic v0.36 with a 4-bp sliding window cutoff of Q30 and minimum sequence length of 80 bp (14). Illumina reads retained after trimming (126 Mb; ~20× coverage) were used to correct indels in medaka-corrected contigs by two iterations of Pilon v1.24 (15). Terminal overlaps on assembled contig were identified manually by sequence identity and contigs circularized using Vim v9.1. Unless stated otherwise, all software were run with default parameters.

*V. harveyi* K2014767 comprises two circular chromosomes (3,610,916 bp and 2,395,843 bp) and 3 plasmids (33,477 bp, 61,846 bp, and 114,494 bp), identified based on sequence homology with previously characterized plasmids using BLASTn. Genome and plasmid sequences were annotated through the NCBI Prokaryotic Genome Annotation Pipeline (PGAP) (16) (Table 1). The Comprehensive Antibiotic Resistance Database’s (CARD) Resistance Gene Identifier (RGI) identified the chromosome-associated *adeF* gene, which encodes the membrane fusion protein of the *adeFGH* multidrug efflux complex associated with tetracycline and fluoroquinolone resistance. Plasmid-associated antibiotic resistance elements were absent (17). PHAge Search Tool Enhanced Release (PHASTER) identified the presence of an intact and incomplete phage element in chromosome 1 and a questionable *Vibrionaceae* family-specific phage element on chromosome 2 (18, 19) .

**TABLE 1:** Genome features for *Vibrio harveyi* isolate K20141767 annotated using PGAP

Genome feature	Chromosome 1	Chromosome 2	Plasmid 1	Plasmid 2	Plasmid 3
Accession no.	CP116414	CP116415	CP116416	CP116417	CP116418
Size	3,610,916	2,395,843	33,477	61,846	114,494
GC content	45%	44.7%	42.1%	42.2%	40.6%
CDS	3,231	2,146	44	67	147
rRNA	34	3			
tRNA	116	16			
ncRNA	3				

## Data Availability

The complete genome sequence of *Vibrio harveyi* isolate K20141767 has been deposited in NCBI under BioProject PRJNA924262 GenBank accession nos. CP116414, CP116415, CP116416, CP116417, and CP116418. The SRAs can be found at SRX23705271, SRX23705272, SRX23705273, SRX23705274, and SRX23705275.
